# An Atlas of the Bacteria Pathogenic in Man

**Published:** 1900-03

**Authors:** 


AN ATLAS OF THE BACTERIA PATHOGENIC IN MAN. With descriptions of their Morphology and Modes of Microscopic Examination. By SAMUEL C. SHATTUCK, F. R. C. S., Joint Lecturer on Pathology and Bacteriology at St. Thomas's Medical School, London ; Pathological Curator of the Museum of the Royal College of Surgeons, London. With an introductory chapter on Bacteriology : Its Practical Value to the General Practitioner. By W. WAYNE BABCOCK, M., D , Fathologist to the Kensington Hospital for Women ; Clinical Pathologist to the Medico Chirurgical Hospital, etc. Sixteen full-page colored plates. New York : E. B. Treat & Co. 1899.

This atlas was originally published in the International Medical Annual in 1898 and 1899 and evoked the admiration of those familiar with bacteriology, that the author and publisher deemed it wise to issue it in book form.

The plates of high artistic merit illustrate the appearance under the microscope of the bacteria pathogenic in the human subject.

All are in colors, handsomely executed with descriptive text, succinct and practical, thus giving a sufficiently complete work on the subject to meet ordinary requirements. An introductory chapter on the practical value of bacteriology to the general practitioner adds to the value of the work. W. C. K.

